# Long term management of obstructive sleep apnea and its comorbidities

**DOI:** 10.1186/s40248-019-0186-3

**Published:** 2019-07-04

**Authors:** Marta Marin-Oto, Eugenio E. Vicente, Jose M. Marin

**Affiliations:** 1 Department of Respiratory Medicine, Clinica Universitaria de Navarra, University of Navarra, Pamplona, Spain; 2 Otorhinolaryngology Service, Hospital Universitario Miguel Servet, Zaragoza, Spain; 3 Respiratory Service, Hospital Universitario Miguel Servet, and Department of Medicine, University of Zaragoza, Avda. Isabel la Católica, 1-3, 50009 Zaragoza, Spain; 4 Traslational Respiratory Research Unit, IISAragon, Zaragoza and CIBER Enfermedades Respiratorias, Instituto Salud Carlos III, Madrid, Spain

**Keywords:** Obstructive sleep apnea, Cardiovascular disease, Hypertension, Diabetes, Dislipemia, Long-term management, Outcomes

## Abstract

Obstructive sleep apnea (OSA) is a worldwide highly prevalent disease associated with systemic consequences, including excessive sleepiness, impairment of neurocognitive function and daytime performance, including driving ability. The long-term sequelae of OSA include and increase risk for cardiovascular, cerebrovascular and metabolic syndrome disorders that ultimately lead to premature death if untreated. To ensure optimal long-term outcomes, the assessment and management of OSA should be personalized with the involvement of the appropriate specialist. Most studies have demonstrated inmediate improvement in daytime somnolence and quality of life with CPAP and other therapies, but the effect of long-term treatment on mortality is still under debate. Currently, the long-term management of OSA should be based on a) identifying physiological or structural abnormalities that are treatable at the time of patient evaluation and b) comprehensive lifestyle interventions, especially weight-loss interventions, which are associated with improvements in OSA severity, cardiometabolic comorbidities, and quality of life. In long-term management, attention should be paid to the clinical changes related to a potential reoccurrence of OSA symptoms and it is also necessary to monitor throughout the follow up how the main associated comorbidities evolve.

## Introduction

We define obstructive sleep apnea (OSA) as an entity characterized by repeated collapses of the pharynx during sleep that reduce or eliminate airflow completely for at least 10 s and in a number of 5 episodes or more every hour of sleep (Apnea-Hipopnea Index, −AHI-). These episodes are associated with sympathetic activation, exaggerated negative swings in intrathoracic pressure, intermittent oxyhemoglobin desaturation, hypercapnia and arousal from sleep. These physiological changes seem to act as intermediate mechanisms responsible for the accelerated development of new comorbidities. This topic has been reviewed extensively in *Multidisciplinary Respiratory Medicine* by MR Bonsignore et al. [1]. In this chapter we will review three relevant questions: 1) the available information on the natural history of OSA and its relationship with incident comorbidities, particularly cardiovascular ones, 2) how the available treatment for patients with OSA does impact the evolution of OSA and 3) how the OSA treatment can modify the health outcomes of the comorbidities associated with OSA. Unfortunately, there is little information in the literature on both subjects. This is so, because since the appearance of continuous positive airway pressure (CPAP) as an effective treatment to reverse OSA symptomatology, it would not be ethical to study the natural history of symptomatic patients with OSA for a long time of period without offering them an effective treatment.

## Clinical course of obstructive sleep apnea

**Fig. 1:**
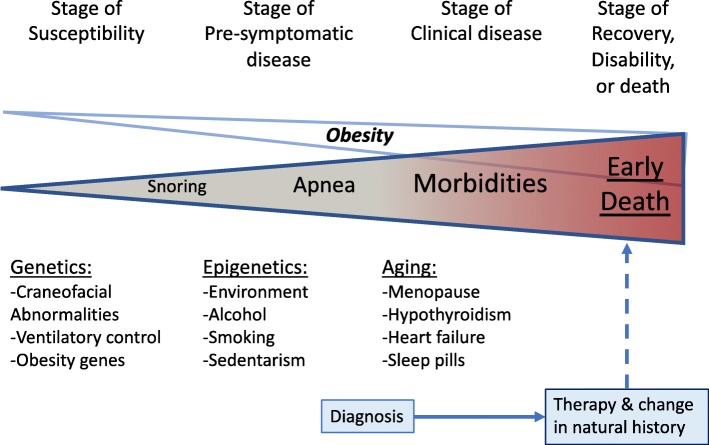
Natural history of obstructive sleep apnea (see text for details)

OSA is actually part of a “continuous” patho-physiological process in which the upper airway (UA), mainly the pharynx, shows a high resistance to air flows (Fig. 1). Initially this dysfunction is asymptomatic or manifested by snoring: “stage of susceptibility”. Predisposed subjects probably have a genetic load of susceptibility that we are largely unaware of. With adulthood and in parallel with weight gain, environmental and epigenetic factors aggravate the collapsibility of the UA. In this “pre-symptomatic” stage, snoring is usually aggravated, nocturnal apneas appear but the subject may not report a diurnal limitation in his/her activities. Without solution of continuity, the patient evolves towards a “stage of clinical illness” in which morbidities develop at younger ages with respect to the non-OSA population in what we could consider in some way an accelerated aging. If patients are not identified and treated, the natural evolution is towards disability and premature death mainly due to cardiovascular events.

In current medicine, knowledge about the natural evolution of diseases is based on the descriptions made by doctors in the last century. It is not possible with current treatments to validate these descriptions through observational cohort studies and much less with randomized controlled trials (RCT). In OSA it happens the same thing. The first detailed descriptions of OSA were made by European authors. However, these reports did not describe the long-term evolution of the disease [2, 3]. Before the description by Sullivan et al of the efficacy of CPAP for the treatment of OSA [4], physicians who treated these patients only had upper airway surgery (including tracheotomy). Early clinical descriptions of OSA included substantial disability and health care utilization, largely reflecting the limited management options available at the time. Many patients evolved towards the development of heart failure and respiratory failure or dying in various accidents. The majority of these patients were young adults. In the last 30 years, many studies on the evolution and short- and long-term management of OSA have been published. We review here briefly the most significant studies grouping them according to their study design: clinical based cohort, community-based cohort and RCT (Table 1).

**Table 1:** Long-term mortality studies in obstructive sleep apnea

1^st^ autor, and reference	Design	Sample	Mean Age	Mean follow up	Results
*Cohort studies published before 1996*					
He et al.; (Ref. #5)	Retrospective	385	52	N. A	AI > 20 has a RR mortality of 1.5 vs AI < 20 UPPP vs conservative: no differences
Partinen et al.; (Ref. #6)	Retrospective	198	56	5 years	Tracheostomy vs conservative 5 years survival: 0% vs 6%
Bliwise et al.; (Ref. #7)	Prospective	298	69	Up 12 years	RR mortality for RDI > 10 of 2.67 (0.95–7.5, 95%CI).
Ancoli-Israel et al.; (Ref. #8)	Prospective	233	83	3.3 years	Significant association of AHI with death in women but not in men
Mant et al.; (Ref. #9)	Prospective	163	83	4 years	No relationship between RDI and survival
Lavie et al.; (Ref. #10)	Prospective	1620	48	12 years	Mortality OR of 1.012 for AI > 10 (not significant)
*Cohort studies published after 1996*					
Lavie et al.; (Ref. #12)	Prospective	13,850	48	4.5 years	HR all-cause mortality, 2.2 for RDI > 30 (significant)
Yaggi et al. (Ref. #13)	Prospective	1022	61	3.4 years	HR all-cause mortality, 3.3 for AHI > 36 (significant)
Marin et al.; (Ref. # 14)	Prospective	1651	50	10 years	OR cardiovascular mortality, 2.87 for AHI > 30 (significant)
Young et al.; (Ref. #15)	Prospective	1522	48	18 years	HR all-cause mortality, 3.8 for AHI > 30 (significant)
Marshall et al.;(Ref. #16)	Prospective	400	53	20 years	HR all-cause mortality, 4.2 for RDI > 15 (significant)
Punjabi et al.; (Ref. #17)	Prospective	6441	63	10 years	HR all-cause mortality, 2.09 for AHI > 30 (in men aged 40–70 years)

*Abbreviations*: *AI* apnea index *RR* risk ratio, *UPPP* uvulopalatopharyngoplasty, *AHI* apnea-hipopnea index, *RDI* respiratory disturbance index, *OD* odds ratio, *HR* hazard ratio, *CVS* cardiovascular, *OSA* obstructive sleep apnea

### Cohort studies

The first clinical-based and retrospective studies seemed to indicate that patients with severe OSA treated with tracheotomy and CPAP had better survival than those treated with uvulo-palato-pharyngoplasty (UPPP) or by conservative measures [5, 6]. Mortality rates were around 6% per 5–8 years among the untreated patients, being cardiovascular events the most frequents causes of death. What was interesting about these early studies is that, despite their methodological limitations, it appears that the “complete” suppression of apneas/hypopneas with treatments such as tracheotomy or CPAP could improve the survival of patients with OSA, while other “partial” efficacy techniques (e.g. the UPPP) did not influence the health outcomes of the patients, therefore cannot be recommended to treat the most severe cases of OSA. There were another four prospective studies with three of them only including elderly population with contradictory results [7–10]. All six studies had many methodologic limitations because they failed adequately to take into account important confounding risk factors for cardiovascular diseases, such as obesity, smoking, dyslipemia, or hypertension. As stated in a systematic review published in 1997 by Wright et al., the results of those studies showed inconsistent results with limited evidence to link OSA with an excess of mortality [11].

Since the Wright's paper was published, many well designed longitudinal studies have confirmed increased mortality in OSA patients. In Israel, Lavie and colleagues collected mortality information among a very large cohort of 14,589 men referred to the sleep clinics with suspected sleep apnea [12]. After a median follow up of 4.6 years, Cox proportional analysis revealed that both BMI and RDI were independently associated with mortality. Unfortunately, no other potential risk of mortality, clinical status at diagnosis, or therapy was controlled. In USA, among patients without pre-existing cardiovascular diseases who were referred to a Sleep Center for the evaluation of sleep-disordered breathing, Yaggi and colleagues reported an increased risk for death or stroke in OSA patients and a dose-effect relationship between OSA severity and risk [13]. Unfortunately, use of nasal CPAP was not evaluated and the short duration of follow up (3 years) and the small number of observed events did not allow the specific assessment of the effects of therapy. In 2005, we reported the long-term cardiovascular outcomes in men with OSA referred to our sleep unit between January 1, 1992, and December 31, 1994 [14]. During the recruitment period, 1,465 patients had polysomnography and treatment with CPAP was recommended to 667 patients. Patients attended the clinic yearly. During these visits, compliance with CPAP therapy was assessed by the timer built into each CPAP device. A mean daily use of more than 4 h per day was considered necessary to maintain the CPAP prescription. After a mean of 10.1 years, Patients with untreated severe OSA had a higher incidence rate of fatal events (1.06 events per 100 person-years) than untreated patients with mild-moderate OSA (0.55 events, < 0.02); simple snorers (0.34 events, *p* < .0005); patients treated with nasal CPAP (0.35 events, *p* < .005); and healthy subjects (0.3 events, *p* < .005). Multivariate analysis adjusted for potential confounders showed that untreated severe OSA increased significantly the risk of fatal cardiovascular events (odds ratio 2.87; 95% CI, 1.17–7.51) compared with healthy subjects (Table 2). At the time, this study was very relevant because it contributed not only to the knowledge of the natural history of OSA, but also to establish AHI > 30 as a defining reference value for severe OSA. It was also the first paper to report that CPAP therapy reduces the risk of fatal and non-fatal cardiovascular outcomes in OSA.

**Table 2:** Fully adjusted odds ratio for cardiovascular death associated to clinical variables and diagnosis status

Variable	Odds Ratio (95% Confidence Interval)	*P*
Age, years	1.09 (1.04–1.12)	0.001
Diagnostic group		
Snoring *(AHI < 5)*	1.03 (0.31–1.84)	0.88
Mild-moderate OSA *(AHI: 5–15)*	1.15 (0.34–2.69)	0.71
Severe untreated OSA *(AHI: 15–30)*	2.87 (1.17–7.51)	0.025
OSA treated with CPAP *(AHI: > 15)*	1.05 (0.39–2.21)	0.74
Prevalent CVS disease	2.54 (1.31–4.99)	0.005

*Abbreviations*: *CPAP* continuous positive airway pressure, *CVS* cardiovascular, *OSA* obstructive sleep apnea, *AHI* apnea-hipopnea index. Adapted from Marin JM, et al. (reference # 14)

Some population studies have confirmed the results of these clinical-based cohort studies. In a 18-year mortality follow up conducted on the Wisconsin Sleep Cohort sample (*n* = 1522), the adjusted hazard ratio (95% CI) for all-cause mortality with severe OSA (AHI > 30) versus no OSA was 3.8 (1.6,9.0) irrespective of symptoms of sleepiness [15]. The Busselton study confirms this finding in a relatively young population in Australia [16], while in a somewhat older population such as Sleep Health Heart Study, the excess mortality associated with OSA was only shown in men [17]. The problem with these three epidemiological studies is that the effect of OSA treatment on health outcomes could not be adequately evaluated.

In addition with mortality, in cohort studies, OSA has been linked with incident cardiovascular outcomes such as hypertension [18], coronary artery disease [19], myocardial infarction [20] and stroke [13]. Given the increased cardiovascular morbidity and mortality in patients with OSA, the possibility that OSA was also a risk factor for developing other cardiovascular risk factors such as diabetes or dyslipidemia, has been investigated in these cohort studies. A recent meta-analysis that includes a total of 64,101 participants reveals that OSA is associated with incident diabetes, with an unadjusted pooled relative risk of 1.62 (95% CI, 1.45–1.80) [21]. There are, however, no reports that have specifically explored the development of dyslipidemia in longitudinal studies. Patients with OSA commonly experience memory problems, and neurocognitive disfunction [22], however, there are no data that allow associating OSA and incident dementia. As part of the cognitive disfunction and daytime sleepiness, it is well known that patients with OSA are at higher risk for motor vehicle accidents [23]. Finally, excess mortality in patients with OSA could also be justified by an increased incidence of all types of malignancies, especially in young adults with severe OSA [24, 25].

### Randomized studies

Long-term randomized controlled trials (RCTs) with the aim of evaluating the effect of treatment on morbidity and mortality in OSA are difficult to carry out due to the insurmountable ethical problems that supposed to stop treating patients with significant diurnal symptoms. However, some RCTs have been carried out to assess the effect of treatment on diurnal symptoms and quality of life during relatively short time. Most studies have evaluated the effect of CPAP on excessive daytime sleepiness (EDS) [26, 27] and health status [27]. Additionally, these studies immediately showed that the positive effects of CPAP required a minimum effective use of more than 4 h a day.

As an alternative for patients intolerant to CPAP, mandibular advancement oral appliance therapy (MAT) can be considered. Some short-term (3 months) RCT have shown similar improvement in somnolence, vigilance, and neurocognitive performance with MAT, compared with CPAP in patients with mild-to-moderate OSA [28]. Upper airway surgery as a treatment option for OSA has been extensively reviewed and meta-analyzed [29], however, until now, RCTs that have demonstrated their efficacy on the symptomatology and quality of life of patients with OSA have not been carried out.

Weight-loss intervention is effective to improve the cardiovascular risk-factor profile in obese patients with or without OSA. In addition, all bariatric surgery procedures achieve improvement in their sleep apnea however, OSA can persist in after substantial weight loss [30] so follow up sleep study needs to be done to determine whether further OSA therapy is needed despite weight loss. In RCT, the combined therapy of CPAP with weight-loss intervention resulted in a larger reduction in blood pressure than either CPAP or weight loss alone [31]. There are no RCT head to head studies to compare the effect of bariatric surgery versus CPAP, MAT or other therapies.

## Role of OSA in the evolution of other comorbid diseases

Given that the majority of patients with OSA present some comorbidity, especially cardiovascular or metabolic, it is relevant to know how the most prevalent and relevant comorbidities, mainly cardiovascular risk factors, will evolve depending on the treatment applied to control apneas.

### Hypertension

Arterial hypertension in patients with OSA should be treated according to the current guidelines regardless of the specific treatment that is to be applied for sleep apnea. Nevertheless, three circumstances must be considered in the relationship hypertension - OSA.

A) In the normotensive patient with OSA who consults for the first time, what is the future risk of developing hypertension? Put it in another way, is the treatment of OSA effective for the primary prevention of hypertension? There are data that suggest it. After considering confounding factors, the odds of developing incident hypertension over 4 years in non-hypertensive OSA patients that received no treatment, was threefold greater for those with an AHI > 15 at baseline in population studies [32], and twofold in clinical studies [33, 34] compared with participants without OSA. However, in the latter study, compared with controls, the adjusted HRs for incident hypertension were greater among patients with OSA ineligible for CPAP therapy (1.33; 95% CI, 1.01–1.75), among those who declined CPAP therapy (1.96; 95% CI, 1.44–2.66), and among those nonadherent to CPAP therapy (1.78; 95% CI, 1.23–2.58), whereas the HR was lower in patients with OSA who were treated with CPAP therapy (0.71; 95% CI, 0.53–0.94) [33]. These results were confirmed in a *post hoc* analysis of a RCT performed during 4 years with normotensive patients with OSA and without excessive daytime sleepiness. In this multicentric study, CPAP treatment reduce the incidence of hypertension or cardiovascular events in patients with CPAP adherence of 4 h/night or longer [35].
B) In patients with OSAS and associated hypertension, how do the blood pressure (BP) figures in treated and untreated subjects behave? This has been one of the most studied topics of sleep medicine related to OSA. From several recent RCTs and meta-analyzes, it can be concluded that: in patients treated with CPAP that show good compliance, diurnal systolic and diastolic BP are reduced by an average of − 2.58 mmHg (95% CI, − 3.57 to − 1.59 mmHg) and − 2.01 (95% CI, − 2.84 to − 1.18 mmHg) compared to patients with untreated OSA. The effects were stronger in younger and sleepier patients and more severe OSA [36]. It should always be borne in mind that the reduction of BP is a collateral effect of CPAP and that, this treatment should not be used with the specific objective of reducing the BP Figs.
C) In a patient with arterial hypertension, when should the co-existence of OSA and its potential role in the pathogenesis of hypertension be suspected? Since more than 80% of OSA patients have a non-dipping BP profiles in a sample of untreated patients with mild to severe OSA [37], hypertensive subjects that demonstrate a BP drop < 10% of daytime values (non-dippers) during a 24 h ambulatory blood pressure monitoring (ABPM), should have a sleep study to rule out OSA. These hypertensive non-dippers are at higher risk of incident cardiovascular events, and increased risk of renal disease progression as compared to nocturnal dippers [38]. Another very important group of hypertensive patients in whom a sleep study is needed to rule out the coexistence of OSA are those with resistant hypertension (RH) defined as an office BP ≥140/90 mmHg despite the use of 3 or more antihypertensive agents [39]. In this subgroup, the prevalence of OSA is reported to be 70–83% [40] and the treatment with CPAP showed a favorable reduction of BP in RCT [41]. In summary, since among hypertensive patients there was a dose-dependent reduction in blood pressure and incident cardiovascular diseases [42], those patients with comorbid OSA who receive effective treatment with CPAP are also receiving a treatment that helps them stabilize their blood pressure and reduce their cardiovascular morbidity and mortality.

### Diabetes

It is recognized that the prevalence of diabetes among patients with OSA is greater than that in the non-OSA population, and exhaustive reviews of the relationship between OSA and diabetes have recently been published [43]. On the other hand, based on clinical and population-based observational cohort studies, patients with severe OSA (eg AHI > 30), without initial diabetes mellitus (DM), are considered to have an increased risk of developing DM in the future. [44, 45]. There is no information on the role played by the long-term treatment of OSA in reducing or not the risk of developing diabetes.

Conversely, in observational studies of diabetic subjects with OSA, effective treatment of OSA tend to improve indicators of glycemic status [46]. A recent systematic review and meta-analysis concluded that CPAP does not improve glycemic control measure as HbA1c [47]. However, the studies reviewed included mostly non-sleepy patients, were of short duration (12 to 24 weeks) and in most of them, the daily use of CPAP was lower than 4 h. Again, the selection of patients with OSA that are included in the RCTs, is in itself a bias that does not reflect the reality of the patients we see in the clinics on a daily basis. For example, it is known that the effect of CPAP on glucose metabolism is more effective when patients are drowsier [48]. The clinician must manage their diabetic patients with OSA based on the clinical guidelines and should focus primarily on weight reduction. as a target treatment for both the management of diabetes and OSA.

### Dyslipidemia

Several observational studies [49] and a meta-regression analysis [50] support the existence of a link between OSA and dyslipidemia. No studies have been conducted to establish whether the treatment or not of OSA is associated with a reduction in the risk of developing dyslipidemia in subjects without lipid alterations at baseline. On the other hand, there are RCTs that have evaluated the response of CPAP in terms of blood lipids in patients with OSA and dyslipidemia with mixed results [51, 52]. Again, it should be emphasized that the results of the RCTs do not exactly reflect the usual patient attended at sleep clinics. For example, the improvement of hypersomnolence could be associated with increased physical activity and caloric output, which can also contribute towards improving dyslipidemia. Therefore, it is difficult to identify in the context of an integral management of the patient with OSA (e.g hygienic-dietetic measures, promotion of exercise, abstinence from tobacco and alcohol, CPAP, upper-airway surgery, etc. ...), which of the therapeutic measures on an individual basis is more effective to improve the lipid profile and health outcomes.

### Cardiovascular diseases

The acute and chronic cardiovascular effects of sleep apnea are well known and have been extensively studied [53]. On the other hand, among patients with OSA, the prevalence of diseases promoted by atherosclerosis (eg, stroke, ischemic heart disease, aneurysms, etc.) is higher. The evidence of an increased risk of cardiovascular morbidity and mortality among patients with untreated OSA is consistent but comes from long-term clinical and population studies [14–17]. There are also epidemiological studies that have indicated a reduction in cardiovascular risk in patients with OSA treated correctly with CPAP or with tracheostomy [14, 54]. The development of RCT studies that confirm a causal relationship will not be possible for reasons indicated above. Based on this evidence and in parallel with how we inform our smokers, the doctor must communicate to their patient with severe OSA the risk and potential benefit of the treatment of their underlying disease.

Another different problem is the influence it has on the clinical course of an already established cardiovascular disease (eg coronary atherosclerotic disease, stroke, aneurysm), suffering from OSA as an associated morbidity. In the cardiovascular literature it is well established that treating, for example, hypertension or dyslipidemia of a patient with established coronary disease will ultimately reduce the likelihood of new cardiovascular events (secondary prevention). The effect of treating OSA in this type of patients is not so clear. RCTs done in patients recruited in cardiac clinics, mostly with already cardiovascular or cerebrovascular events, did not show an improving in morbi-mortality compared to those treated with CPAP. Nevertheless, a significant improvement was reported in daytime sleepiness, quality of life, mood, and work productivity in patients who received CPAP [55, 56]. From the practical point of view and until we know the results of more RCT currently underway, we must act with patients with cardiovascular disease and suspected OSA, following the same strategy as with the “non-cardiovascular” patients. That is, based on a good sleep history, ordering the appropriate sleep study and designing the personalized treatment for each case based on the current guidelines. For our part, we would add that the sleep studies in this type of patients should always be “attended” to specify the dominant type of breathing-sleep disorder (eg, obstructive apneas, central apneas) and if positive pressure ventilation is required, its titration should always be done manually in a second sleep study.

## The present strategy in the long-term management of OSA

**Fig. 2:**
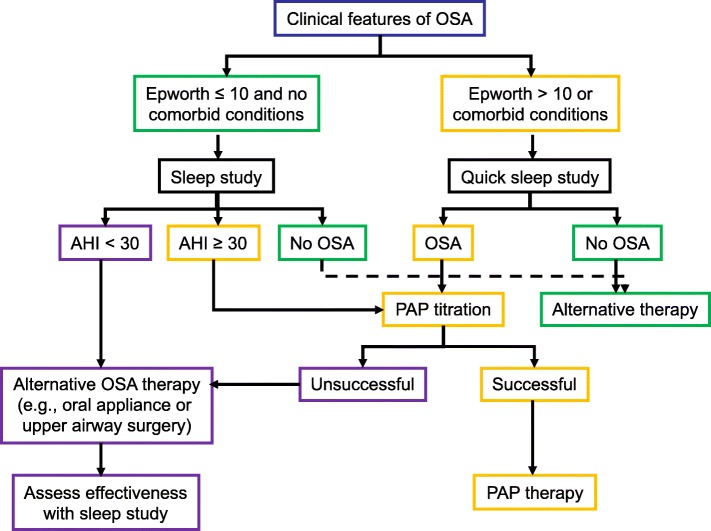
Treatment algorithm for obstructive sleep apnea (OSA). This flow diagram shows a general approach to the management of patients with suspected OSA. See Box 61–2 for the Epworth Sleepiness Scale. AHI, apnea-hypopnea index; PAP, positive airway pressure

There is no worldwide consensus about the management of OSA. Several scientific societies have clinical management guidelines for the initial treatment of OSA [57–59]. Figure 2 shows our strategy for the prescription of CPAP. Currently sleep specialist are moving to treat their patients from a mechanistic perspective grouping patient into phenotypic traits that cause OSA such upper airway anatomic compromise, high loop gain, low respiratory arousal threshold and poor pharyngeal muscle responsiveness during sleep [60]. However, there is no specific recommendation on how the patient’s long-term follow up process should be, what specialist should initiate the patient’s diagnostic and therapeutic process, how often and until when a patient’s course should be followed after being diagnosed or when should a new sleep study be carried out.

In addition to an intervention to increase the upper airway lumen with pharyngeal surgery or to prevent the collapsibility of the upper airway with the application of CPAP, the management of OSA should always include lifestyle intervention. A comprehensive lifestyle intervention (CLI) program includes a reduced-calorie diet, exercise/increased physical activity, and behavioral counseling. A seminal RCT of a CLI demonstrated significant improvement in AHI parallel with weightloss [61]. CLI is particularly effective in overweight and obese patients with OSA. A CLI program that effectively achieves a weight reduction, not only improves the AHI, but simultaneously impacts on the prognosis of a coexisting diabetes [62], hypertension and cardiovascular diseases [63]. A recent Clinical Practice Guideline document from the American Thoracic Society summarizes the principles and recommendations of CLI in the management of OSA [64].

The current trend is that any recommendation included in the guidelines must be strictly evidence-based. However, in many real-world situations, evidence is not available. In our opinion, when irrefutable evidence is lacking on certain aspects of clinical management, common sense and good practices should prevail. Some recommendations in the field of sleep-breathing disorders should be implemented without the need for large randomized trials. Indeed, there is no randomized trial supporting the benefit of smoking cessation, yet it is recommended in all guidelines. One of the responsibilities of a physician is to interpret their patients’ individual problems. We have brought in our Sleep Clinic the recommendations derived from scientific knowledge in the area of OSA, and, following the worthy example of a passage from the Old Testament that has served humanity for thousands of years, have set them down in 10 OSA commandments (Table 3). This simple guideline constitutes an attractive, easy and practical approach to the management of COPD in all its variations and will give physicians the freedom to provide the best possible care to their patients.

**Table 3:** The 10 OSA Commandments®

Prevention	I	Help to avoid overweight and obesity
Diagnosis	II	Suspect OSA in cases of snoring and/or visualized apneas or daytime sleepiness
	III	Confirm the diagnosis. Perform sleep study
	IV	Quantify sleepiness, BMI, AHI, and nocturnal hypoxemia
	V	Identified comorbidities, particularly cardiovascular, metabolic and neurocognitive deficits
	VI	Explore the upper airway and look for treatable anatomic abnormalities
Treatment	VII	Establishes a program to reduce weight
	VIII	Start patient-specific treatment
	IX	Supervise the correct use of CPAP and other devices and medications
Follow-up	X	Establish a follow up plan and measure response to treatment

*Abbreviations*: *OSA* obstructive sleep apnea, *BMI* body mass index, *AHI* apnea-hipopnea index, *CPAP* continuous positive airway pressure

*Copyright© 2016. Grupo BODE*

## Conclusions

The current knowledge about the clinical course or natural history of the disease in the case of obstructive sleep apnea, comes largely from the clinical experience of the physicians who have handled this type of patients for decades. In support of this knowledge, we only have some clinical-base and populational observational studies. Unlike other areas of medicine, in the case of OSA, we will not be able to have large long-term RCTs that help us to define the management of our patients. At present, the initial treatment of patients with OSA should focus on eliminating apneas with personalized therapy for each subject with the ultimate goal of normalizing the quality of life and control or delay the occurrence of comorbidities. To help achieve this goal, we must include the patient in lifestyle improvement programs with the ultimate goal of reducing weight and increasing physical activity, especially in overweight or obese subjects.

## Abbreviations

ABPM: Ambulatory blood pressure monitoring
AHI: Apnea hypopnea index
BMI: Body mass index
BP: Blood pressure
CPAP: Continuous positive airway pressure
DM: Diabetes mellitus
MAT: Oral appliance therapy
OSA: Obstructive sleep apnea
RCT: Randomized controlled trial
RDI: Respiratory disturbance index
RG: Resistant hypertension
UA: Upper airways
UPPP: Uvulopalatopharyngoplasty
